# The effect of low birth weight as an intrauterine exposure on the early onset of sarcopenia through possible molecular pathways

**DOI:** 10.1002/jcsm.13455

**Published:** 2024-03-29

**Authors:** Dilek Celik, Manuela Campisi, Luana Cannella, Sofia Pavanello

**Affiliations:** ^1^ Department of Pharmceutical and Pharmacological Sciences University of Padua Padua Italy; ^2^ Department of Cardiac Thoracic Vascular Sciences and Public Health University of Padua Padua Italy; ^3^ University Hospital of Padova Padua Italy

**Keywords:** Clock genes, DOHaD, Epigenetic, Genome‐wide study, Low birth weight, Sarcopenia

## Abstract

Sarcopenia, a musculoskeletal disease characterized by the progressive loss of skeletal muscle mass, strength, and physical performance, presents significant challenges to global public health due to its adverse effects on mobility, morbidity, mortality, and healthcare costs. This comprehensive review explores the intricate connections between sarcopenia and low birth weight (LBW), emphasizing the developmental origins of health and disease (DOHaD) hypothesis, inflammatory processes (inflammaging), mitochondrial dysfunction, circadian rhythm disruptions, epigenetic mechanisms, and genetic variations revealed through genome‐wide studies (GWAS). A systematic search strategy was developed using PubMed to identify relevant English‐language publications on sarcopenia, LBW, DOHaD, inflammaging, mitochondrial dysfunction, circadian disruption, epigenetic mechanisms, and GWAS. The publications consist of 46.2% reviews, 21.2% cohort studies, 4.8% systematic reviews, 1.9% cross‐sectional studies, 13.4% animal studies, 4.8% genome‐wide studies, 5.8% epigenome‐wide studies, and 1.9% book chapters. The review identified key factors contributing to sarcopenia development, including the DOHaD hypothesis, LBW impact on muscle mass, inflammaging, mitochondrial dysfunction, the influence of clock genes, the role of epigenetic mechanisms, and genetic variations revealed through GWAS. The DOHaD theory suggests that LBW induces epigenetic alterations during foetal development, impacting long‐term health outcomes, including the early onset of sarcopenia. LBW correlates with reduced muscle mass, grip strength, and lean body mass in adulthood, increasing the risk of sarcopenia. Chronic inflammation (inflammaging) and mitochondrial dysfunction contribute to sarcopenia, with LBW linked to increased oxidative stress and dysfunction. Disrupted circadian rhythms, regulated by genes such as BMAL1 and CLOCK, are associated with both LBW and sarcopenia, impacting lipid metabolism, muscle mass, and the ageing process. Early‐life exposures, including LBW, induce epigenetic modifications like DNA methylation (DNAm) and histone changes, playing a pivotal role in sarcopenia development. Genome‐wide studies have identified candidate genes and variants associated with lean body mass, muscle weakness, and sarcopenia, providing insights into genetic factors contributing to the disorder. LBW emerges as a potential early predictor of sarcopenia development, reflecting the impact of intrauterine exposures on long‐term health outcomes. Understanding the complex interplay between LBW with inflammaging, mitochondrial dysfunction, circadian disruption, and epigenetic factors is essential for elucidating the pathogenesis of sarcopenia and developing targeted interventions. Future research on GWAS and the underlying mechanisms of LBW‐associated sarcopenia is warranted to inform preventive strategies and improve public health outcomes.

## Introduction

As the world rapidly ages, sarcopenia emerges as a global public health concern that requires prioritized attention from researchers and practitioners. Sarcopenia is a progressive skeletal muscle disorder characterized by rapid loss of excessive muscle mass, strength, and physical performance.^1^ It brings along severe adverse health consequences such as deteriorated mobility, frailty, falls, increased morbidity and mortality, and high healthcare costs,^1^, ^2^ and accordingly has a negative impact on quality of life.^3^ This complex disease involves various risk factors, encompassing environmental and genetic influences, leading to diverse developmental pathways.^4^

Recent research has shed light on factors impacting foetal growth and development, with a specific focus on prenatal exposure such as low birth weight (LBW), which carries lasting consequences into later life.^5^ Aligning with the Developmental Origin of Health and Disease (DOHaD) theory, maternal metabolism and prenatal dietary habits influence the likelihood of adult disorders through early‐life epigenetic modulation of gene expression.^6^ Promoting healthy early‐life behaviours during pregnancy and infancy and identifying intrauterine exposures and early‐life predictors offer opportunities for preventing later‐life diseases.^6^, ^7^ Investigating the early stages of muscle growth and alterations is crucial for understanding age‐related muscular diseases like sarcopenia.^8^

This comprehensive review aims to explore recent literature on sarcopenia, emphasizing current and potential determinants, underlying mechanisms (including inflammaging, circadian disruption, and mitochondrial dysfunction), and the epigenetic approach. A particular focus is placed on investigating the hypothesis of LBW as a potential early predictor of sarcopenia development.

## Search strategy and selection criteria

We developed a search strategy in PubMed for publications in English using search terms ‘sarcopenia’ and ‘low birth weight’ in combination with one of the following keywords: ‘developmental origins of disease and health’, ‘inflammaging’, ‘mitochondrial dysfunction’, ‘clock genes’, ‘epigenetic’, ‘genome‐wide meta‐analysis’. Since LBW is a main consideration for the development of sarcopenia in our review, we looked into the keywords used for sarcopenia and also for ‘low birth weight’ (Table 1). The publications consist of 46.2% reviews, 21.2% cohort studies, 4.8% systematic reviews, 1.9% cross‐sectional studies, 13.4% animal studies, 4.8% genome‐wide studies, 5.8% epigenome‐wide studies, and 1.9% book chapters.

**Table 1:** Article numbers for sarcopenia and presented key words

Specific term	Following keywords	Article numbers	Included articles
Sarcopenia	**Developmental origins of health and disease**	*N* = 3	1
	‐ Epidemiology	*N* = 161	10
	**Low birth weight**	*N* = 9	8
	‐ DOHaD	*N* = 48	6
	‐ Muscle strength	*N* = 71	11
	**Inflammaging**	*N* = 11	3
	‐Chronic inflammation	*N* = 439	17
	**Mitochondrial dysfunction**	*N* = 350	8
	‐Low birth weight	*N* = 37	4
	**Clock genes**	N = 7	4
	‐Circadian rhythm	*N* = 17	6
	‐Circadian disruption	N = 9	3
	‐Low birth weight	*N* = 31	2
	‐BMAL1 gene	N = 3	2
	‐BMAL1 gene and adiposity	*N* = 63	2
	**Epigenetic**	*N* = 72	5
	‐DOHaD	*N* = 199	7
	‐Histone modification and low birth weight	N = 7	2
	**Genome‐ wide meta‐analysis study**	*N* = 5	4

## Sarcopenia and developmental origins of health and disease theory

The DOHaD hypothesis, pioneered by Prof. David Barker and colleagues, proposes a link between intrauterine exposures, considering also the Dutch Famine study, and the early onset of metabolic diseases in adulthood.^9^ This theory suggests that a mismatch between the intrauterine environment and the predicted postnatal conditions can lead to metabolic impairments later in life.^10^ In a seminal study conducted by Sayer et al.,^11^ a retrospective cohort analysis in Hertfordshire, UK, focused on exploring the associations between birth weight, weight at 1 year of age, and body composition in older men born between 1931 and 1939. The study revealed a positive correlation between birth weight and adult body mass index (BMI) and fat‐free mass, but not with fat mass. Conversely, weight at 1 year of age correlated with BMI, fat‐free mass, and fat mass. These findings suggest that prenatal and maternal influences may predominantly impact fat‐free mass rather than fat mass in older individuals, while postnatal factors could play a more significant role in later obesity development.

The concept of DOHaD considers LBW as a marker of the in utero environment and negative health outcomes in adulthood,^12^ and it has been explored largely through epidemiological and clinical studies. Growing data from research also shows that prenatal life plays a significant role in the early development of adult health, particularly in long‐term conditions related to chronic inflammation and oxidative stress,^13^ which are highlighted in the underlying mechanisms of metabolic diseases and the ageing process.

The DOHaD theory is regarded as a crucial approach in muscle/fat distribution‐based foetal development through epigenetic modifications.^13^, ^14^ Epigenetic changes such as DNA methylation (DNAm), histone modifications, and non‐coding microRNAs in the fetus may cause metabolic diseases later in life since the epigenome is especially vulnerable to changes during the prenatal period due to the high rate of DNA synthesis and DNAm in tissue and organ development during gestation.^9^, ^13^, ^15^ Also, it has already been confirmed with an epigenome‐wide study that there is a correlation between birth weight and DNAm alterations, which remain through adulthood, and this link influences the ageing process: individuals with lower birth weight have accelerated cellular ageing.^12^ Therefore, the DOHaD findings are crucial for the biological plausibility between epigenetic modifications and later life diseases and provide critical public health implications since foetal growth disturbances and body composition imbalances have a significant effect on the early programming of metabolic diseases.

## Sarcopenia and low birth weight

The World Health Organization (WHO) classifies LBW as less than 2500 g, and LBW infants are 20 times more likely to experience problems than normal weight infants.^16^ Therefore, WHO highlights LBW as a critical public health issue.^17^ According to the DOHaD approach, LBW is often triggered by intrauterine exposures and is associated with chronic disease in later life.^18^, ^19^, ^20^, ^21^ The concept of DOHaD focuses on body mass at birth, foetal origins of diseases and early life exposure effects on the development of metabolic disorders in early adulthood.^22^, ^23^ Also, considering muscle comprises 25% of the body composition at birth, early programming of body composition during foetal development, and other epigenetic modifications in the prenatal period, LBW may be deemed a risk factor for early sarcopenia onset regarding epigenetic regulation.

One of the first indication on birth weight associated with sarcopenia in men and women, independently of adult height and weight was published by Sayer et al.^24^ LBW is associated with decreased muscle mass, muscle and grip strength, and lean body mass in early adulthood.^7^, ^24^, ^25^, ^26^, ^27^, ^28^, ^29^, ^30^, ^31^, ^32^, ^33^, ^34^ Also, a lower muscle fibre score was found in the vastus lateralis muscle of old men who were with LBW.^26^ The number of muscle fibres is considered a significant factor in determining muscle strength and mass,^7^, ^35^ which form and grow dramatically throughout gestation and the first year following birth.^8^ The growth of fewer muscle fibres during the intrauterine period may result in early progressive loss of muscle with age, limiting physical ability and independence in early adulthood.^30^ Furthermore, LBW combined with rapid postnatal development results in increased skeletal muscle ageing. DOHaD considers that early adiposity and body composition programming prepares the foetal epigenome for the postnatal era.^36^ When there is a mismatch between the predicted and exposed environments (i.e., malnutrition or low calorie intake),^37^ infants with LBW have a higher fat percentage and lower lean body mass, leading to an increased risk of metabolic disorders such as sarcopenia later in life.^31^, ^36^, ^38^ In light of this knowledge, LBW might be regarded as a marker of intrauterine conditions,^30^ and the effects of prenatal exposures on muscle morphology and adipose tissue formation may be the link between LBW and muscle‐related disorders like sarcopenia.^26^

Moreover, studies have demonstrated that prenatal nutrition is critical for birth weight^39^, adiposity,^36^ and muscle fibre number.^26^, ^40^ LBW and prenatal malnutrition are linked to a higher risk of developing sarcopenia than normal birth weight in adulthood.^7^ Animal and human models have shown that undernutrition during the intrauterine period affects myofiber growth and birth weight, and lower myofibers have negative impacts on muscle mass and grip strength in later life.^26^, ^41^, ^42^ Furthermore, diet during the intrauterine period affects the methylation process in the foetus epigenome, which is supported by epigenetic studies showing that LBW is related to epigenetic alteration in DNAm due to adverse intrauterine exposures.^26^, ^39^

## Sarcopenia and inflammaging

Inflammaging, or chronic low‐grade inflammation that occurs as part of the ageing process, emerges as a key contributor to the development of sarcopenia. This association is grounded in factors such as genetic susceptibility, cellular senescence, and oxidative stress resulting from disruptions in mitochondrial function.^43^ The prevalence of inflammaging, not only leads to tissue impairment independently of infection but also serves as a crucial link connecting age‐driven increases in adiposity, metabolic imbalances, and the onset of sarcopenia and subsequent muscle weakness.^44^, ^45^

### Inflammatory mediators and their impact

Imbalances in reactive oxygen species (ROS) production, attributed to the rise in inflammatory markers like tumour necrosis factor alpha (TNFα), interleukin 6 (IL‐6), IL‐12, nuclear factor kappa B (NF‐κB), and C‐reactive protein (CRP), are intrinsically tied to the ageing process. These mediators activate diverse transcription factors that influence gene expression, ultimately leading to the loss of muscle mass and strength—central to sarcopenia.^46^, ^47^, ^48^, ^49^, ^50^ The inflammaging, referred to as low‐grade inflammation, is associated with an increase in the number of cells that complete the cell cycle and reach the status of cellular senescence, which is a hallmark of ageing.^46^, ^51^ Furthermore, inflammaging can lead to telomere and telomerase impairments, prompting the ageing process, where critically short telomeres denote irreversible DNA damage and consequent cellular senescence.^46^

### Sarcopenia, inflammation, and disease

Sarcopenia is identified by skeletal muscle inflammation and involves molecular impairments associated with various chronic diseases characterized by significant mitochondrial dysfunction and circadian rhythm disruption.^52^ These observations suggest a clear interaction between inflammatory mediators and muscle mass, impacting the development of sarcopenia.^53^

### Muscle and insulin metabolism

Skeletal muscle plays an important role in insulin‐induced glucose metabolism. Notably, the reduction in muscle mass, a hallmark of sarcopenia, is closely attributed to insulin resistance.^54^ Intrauterine growth restrictions leading to LBW contribute to the development of insulin sensitivity in skeletal muscle.^55^ A strong association between TNFα and insulin resistance further underlines the complex relationship.^56^ Population‐based data show that sarcopenic individuals have elevated plasma levels of IL‐6, CRP, and TNFα and their association with an increased risk of muscular strength loss.^46^ It also explains the later phenotype of metabolic syndrome in adults, such as sarcopenia, which is caused by inflammatory biomarker‐related skeletal muscle changes.^56^

### Low birth weight and inflammation

Epidemiological studies have demonstrated the relationship between LWB and increased adulthood risk for cardiovascular^57^, ^58^ and metabolic diseases,^59^, ^60^, ^61^ which could be mediated by an inflammatory pathway. Wada et al. demonstrated that LBW was related to elevated white blood cell counts independently of sex, age, lifestyles, and chronic diseases in middle‐aged Japanese men and women.^62^

In summary, the complex connection between inflammaging and sarcopenia involves a blend of genetic, cellular, and molecular processes. The impact of inflammatory mediators on muscle health cannot be underestimated, as evidenced by their role in the development of sarcopenia. Understanding this interaction is crucial for developing interventions to mitigate age‐related muscle loss and its associated functional impairments.

## Sarcopenia and mitochondrial dysfunction

Recent studies have connected LBW to mitochondrial dysfunction and an excess of ROS, as well as demonstrating that LBW is most likely caused by an increased mitochondrial DNA (mtDNA) copy number in maternal blood.^16^ An increased mtDNA copy number has been determined to be a potentially efficient predictor of LBW and intrauterine growth restriction.^16^ Changes in mtDNA copy number can result in lower performance for electron transport chain (ETC) function and higher ROS generation, which seems connected to the Bcl‐2/adenovirus E1B 19‐kDa‐interacting protein 3 (BNIP3) function. BNIP3, a pro‐apoptotic mitochondrial protein from the Bcl‐2 family, exists in different organs and the placenta in humans.^16^ Decreased expression of muscle BNIP3 induces accelerated ageing and muscle atrophy, which are associated with the development of sarcopenia, compared to high levels of BNIP3 in aged subjects.^63^, ^64^

Mitochondrial activity and organization are crucial factors for skeletal muscle mass and functionality, and inflammatory cytokines such as TNFα and IL‐6 lead to mitochondrial dysfunction during ageing.^65^ Mitochondrial dysfunction in muscles has been reported to have a role in the pathophysiology of sarcopenia, which involves mtDNA depletion, ETC damage, and oxidative stress in aged muscles.^66^ As mitochondrial activity deteriorates, the ROS process is disturbed, resulting in an adverse change in cellular functioning that induces oxidative stress, resulting in pathological consequences in muscle.^67^, ^68^ Anomalies in mitochondrial functioning and errors have also been connected to senescence.^69^ Also, muscle mitochondrial dysfunction linked to intrauterine undernutrition and LBW results in insulin resistance which may cause metabolic problems in skeletal muscle functioning, leading to sarcopenia.^65^, ^70^, ^71^

Furthermore, the accumulation of mtDNA mutations correlates with a decrease in energy generation in muscle cells, weakness, and fibre loss.^72^ mtDNA mutations increase with age and cause mitochondrial dysfunction and skeletal apoptosis, which are major contributions to the pathophysiology of sarcopenia.^73^ Therefore, mitochondrial impairment is considered the key precursor of the underlying mechanism of sarcopenia^74^ and one of the explanatory elements of the link between LBW and sarcopenia.

## Sarcopenia and clock genes: A comprehensive exploration

Clock genes, integral components of the circadian rhythm system, play a crucial role in regulating diverse physiological processes. This discussion delves into the intricate relationship between clock genes and sarcopenia, shedding light on how disruptions in circadian rhythms can influence foetal development, birth weight, metabolic regulation, and the onset of sarcopenic traits.

### Circadian rhythm and foetal development

Disrupted biological clocks, leading to chrono disruption, exert a deleterious impact on foetal development and birth weight.^75^ Moreover, a correlation exists between LBW and adult cortisol levels, a marker of circadian rhythm, indicating LBW's potential to predict prospective metabolic dysregulations.^76^ Animal studies also confirmed that maternal circadian arrhythmia during pregnancy may elevate the risk of developing chronic diseases later in life, aligning with research on DOHaD.^77^

### Circadian regulation and metabolism

The circadian clock's role is pivotal in synchronizing an organism's metabolism with its external environment.^78^ Brain and muscle ARNT‐like protein (BMAL1) and circadian locomotor output cycles kaput (CLOCK) are key transcription factors driving circadian rhythm regulation. Deficiencies in BMAL1 can result in abnormalities in behavioural and genetic expression patterns.^78^ BMAL1 also governs adipogenesis, and circadian disruption impacts lipid metabolism, leading to metabolic disorders in early life.^79^

### Clock genes and sarcopenia

Infants and adults with LBW show visceral adipose tissue accumulation, resulting in reduced lean body mass.^22^ Furthermore, disruption of BMAL1 function in visceral adipose tissue is observed in patients with metabolic diseases.^80^ Rev‐Erbα, a nuclear receptor, regulates adiposity and BMAL1 transcriptional control, influencing myogenic progenitor proliferation and formation.^81^, ^82^ In human models, CLOCK and BMAL1 transcription factors are associated with body weight.^83^ Mice with BMAL1 mutations exhibit smaller body weight and higher triglyceride levels in skeletal muscles.^84^ Therefore, the interaction of the impaired BMAL1 gene, Rev‐Erbα, and LBW may contribute to sarcopenia development. Further research into the circadian rhythm's effect on lipid metabolism and fat/muscle distribution in the intrauterine and postnatal periods could yield significant insights.

### Circadian rhythm disruption and sarcopenia

Recent studies reveal that circadian rhythm disruption from shift work or nocturnal lifestyles contributes to sarcopenia development via molecular circadian clock impairment and mitochondrial dysfunction.^85^, ^86^ BMAL1 regulates homeostasis by controlling ROS; thus, its dysfunction is linked to excessive ROS generation, leading to chronic oxidative stress.^78^ BMAL1 deficiency results in muscular atrophy, decreased strength, altered sarcomere organization, and reduced mitochondrial content—key features of sarcopenia.^81^ Animal models suggest that BMAL1 deficiency shortens lifespan, accelerates ageing, and triggers early‐onset sarcopenia.^78^, ^87^, ^88^

### Clock genes, telomere dynamics, epigenetics, and sarcopenia

CLOCK deficiency in animals correlates with decreased telomerase activity and shorter telomere length—a critical aspect of biological ageing.^89^ Therefore, clock gene disruption is associated with ageing.^90^ Even though the connection between telomere length and sarcopenia is not fully elucidated, shorter telomere length is linked to decreased grip strength—a sarcopenia marker.^91^ Furthermore, epigenetic mechanisms such as histone alterations, including acetylation and methylation, play a role in regulating CLOCK transcription factor expression.^92^ CLOCK has histone acetyltransferase function, and several epigenetic modification enzymes follow circadian rhythm patterns.^92^ Thus, circadian clock disturbances may contribute to LBW and early‐stage sarcopenia through epigenetic modifications.

The influence of clock genes on foetal development, metabolic regulation, and sarcopenia onset is intricate and multifaceted. Understanding how circadian rhythm disruptions affect various life stages provides insights into potential interventions to mitigate sarcopenia and age‐related traits.

## Sarcopenia and epigenetic

Epigenetic alterations, encompassing inheritable gene expression regulation elements, have profound implications for health across generations.^36^ This section explores the complex mechanism between epigenetic mechanisms and the development of sarcopenia, focusing on DNAm, histone modifications, and noncoding RNA regulation. Key epigenetic processes, namely DNAm, histone modifications, and noncoding RNA regulation, play pivotal roles in regulating gene expression.^93^

### Epigenetic mechanisms and developmental origins of health and disease theory

Epigenetic mechanisms serve as the foundation for the DOHaD theory, which states that prenatal and early‐life experiences shape long‐term health outcomes and supports the connection between LBW and increased risk of chronic diseases in adulthood due to the mismatch concept.^93^

### DNA methylation and sarcopenia

DNAm emerges as a major epigenetic mechanism in the context of DOHaD, influencing foetal programming and development.^36^ An epigenome‐wide study on a cohort of 1757 individuals has shown a significant correlation between birth weight and blood‐based DNAm in adulthood.^12^ This study has also reported significant associations between lower birth weight, and higher Grim Age acceleration and shorter DNAm‐derived telomere length, which are two epigenetic age measures, confirming previous results.^94^, ^95^ DNAm is also implicated in the underlying processes of sarcopenia, affecting satellite cell differentiation during early life and the loss of myogenic capacity in ageing.^96^ Peterson et al. found that DNAm mediates the link between chronic diseases and lower grip strength, which has been designated as an important predictor of sarcopenia.^45^ DNAm is reversible, and ten‐eleven translocation methylcytosine dioxygenases (TET) enzymes can demethylate DNA.^36^ Prenatal exposures may disrupt TET activity and be linked to developmental issues.^36^ Animal studies have linked TET mutations to low body mass in mice and developmental issues due to disturbed TET activity.^36^, ^93^

Further investigation is necessary to comprehend how foetal stressors lead to DNAm alterations at specific loci, elucidating the potential connection between early‐life LBW exposure and later‐life sarcopenia.

### Histone modifications and epigenetic interactions in the onset of sarcopenia

Histone tails undergo various modifications, including methylation, acetylation, and phosphorylation, with DNAm and histone modifications engaging in intricate epigenetic interactions.^93^ These modifications are linked to metabolic disorders.^97^ Histone acetylation identifies transcriptionally active genes, while trimethylation of lysine 27 (H3K27me3) and 9 (H3K9me3) indicates silenced genes or regions on histone H3; increased H3K27 methylation levels negatively impact muscle regeneration.^93^ In addition, animal studies establish connections between LBW and increased gene acetylation and H3K9 trimethylation, genes involved in developmental processes and critical for physiological and cellular homeostasis.^98^ Non‐coding RNAs also contribute to the complex network of gene regulation through DNAm.^93^

As epigenetic research advances, it appears that DNAm patterns may serve as markers for early‐life exposures, potentially predicting the likelihood of disorders such as sarcopenia later in life. Within the DOHaD framework, epigenetic alterations play a crucial role in the ageing process and the early onset of chronic disorders.^36^ The exploration of epigenetic mechanisms provides insights into potential interventions to mitigate the development of age‐related conditions, emphasizing the importance of considering early‐life experiences in the context of lifelong health.

## Sarcopenia and genome‐wide studies

Genome‐wide studies (GWAS) are critical to identifying a connection between sarcopenia and possible exposure effects through genes. A GWAS identified the fat mass and obesity‐associated (FTO) gene as a candidate for lean body mass^99^ and found that lower lean body mass relates to sarcopenia. It is also indicated that thyrotropin‐releasing hormone receptor (TRHR), hypothalamic–pituitary‐thyroid (HPT), insulin‐like growth factor‐I (IGF1), and iroquois homebox gene 3 (IRX3) are significant genes for lean body mass determinants.^100^ Thus, there is a gene–gene interaction indicating a link between FTO, IGF‐1, and IRX3, implying that FTO may play an important role in muscle development.^99^ Muscle weakness GWAS results, as another important determinant of sarcopenia, were associated with major histocompatibility complex, class II, DQ Alpha 1 (HLA‐DQA1), growth/differentiation factor 5 (GDF5), and dymeclin (DYM) variants, and the analysis results pointed out a causal link between several chronic disorders and muscle weakness.^101^ Human leukocyte antigen (HLA) alleles were also linked to sarcopenia and its determinants.^102^ Furthermore, in a meta‐analysis of the DNAm genome‐wide study, candidate genes, DNAm modifications (hypo and hyper‐methylations), and gene expression variations with age were identified in skeletal muscle atrophy, lipid metabolism, and fibre type specification.^103^ Therefore, it may be possible to elaborate on the gene loci and biological plausibility of LBW's effect on sarcopenia with GWAS aimed at determining the pathways by which loci play a role in disorders^104^ (Table 2).

**Table 2:** Genomic loci with genes closest to sarcopenia‐related traits

SNP	Nearest gene	Chr/allele	Trait	*P*‐value	Subjects	References
rs9936385	FTO	16/C/T	Low lean body mass, low muscle mass and low body size	6.12 × 10^−12^	2207 in GWAS 44 296 in replication samples (Caucasians)	^99^
rs34415150	HLA‐DRB1 HLA‐DQA1	6/G	Low muscle strength, low grip strength	4.4 × 10^−17^	256 223 for meta‐analysis (European), the UK Biobank, the US Health and Retirement Study, the Framingham Heart Study, and others.	^101^
rs143384	GDF5	20/A	Low muscle strength, low grip strength	4.5 × 10^−13^	256 223 for meta‐analysis (European), the UK Biobank, the US Health and Retirement Study, the Framingham Heart Study, and others.	^101^
rs62102286	DYM	18/T	Low muscle strength, low grip strength	5.5 × 10^−11^	256 223 for meta‐analysis (European), the UK Biobank, the US Health and Retirement Study, the Framingham Heart Study, and others.	^101^
rs13107325	SLC39A8	4/T	Low grip strength	4.4 × 10^−23^	256 223 for meta‐analysis (European), the UK Biobank, the US Health and Retirement Study, the Framingham Heart Study, and others.	^101^
rs9268645	HLA (HLA‐DQA1*03:01, HLA‐DRB1*04:04, HLA‐DQA1*01:02)	6/G/C	Sarcopenia, low grip strength, type 1 diabetes	1.50 × 10^−6^	451 447 (European), UK Biobank	^102^

Genome‐wide significance *P* < 5 × 10^−8^.

DMY, dymeclin; FTO, fat mass and obesity associated gene; GDF5, growth/differentiation factor 5; HLA, human leukocyte antigen; SLC39A8, solute carrier family 39 member 8.

## Summary and conclusions

In summary, our review explored the intricate connections between sarcopenia and LBW through a PubMed search, shedding light on the multifaceted nature of this condition. The review identified key factors contributing to sarcopenia development, including the DOHaD hypothesis, LBW's impact on muscle mass, inflammaging, mitochondrial dysfunction, the influence of clock genes, the role of epigenetic mechanisms, and genetic variations revealed through GWAS.

**Figure 1:**
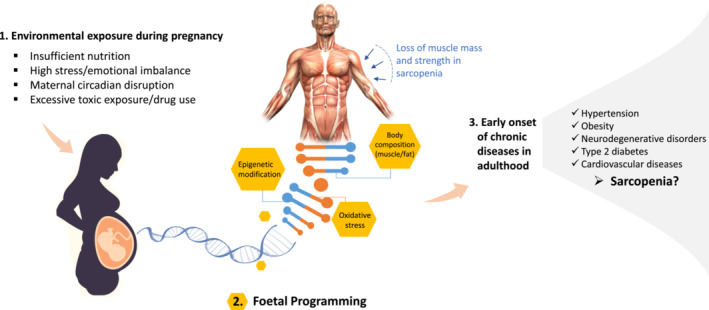
Early‐life exposures and foetal programming: Implications for sarcopenia and long‐term health outcomes.

The DOHaD hypothesis suggests that early‐life exposures, such as LBW, can induce epigenetic alterations during foetal development, impacting long‐term health outcomes, including the early onset of sarcopenia (Figure 1). LBW correlates with reduced muscle mass, grip strength, and lean body mass in adulthood and an increased risk of sarcopenia later in life. Chronic inflammation (inflammaging) and mitochondrial dysfunction both contribute to sarcopenia, with LBW linked to increased oxidative stress and dysfunction. Disrupted circadian rhythms, regulated by genes known as BMAL1 and CLOCK, are linked to both LBW and sarcopenia, impacting lipid metabolism, muscle mass, and ageing processes. Early‐life exposures, including LBW, can induce epigenetic modifications such as DNAm and histone changes, which play a pivotal role in sarcopenia development. Genome‐wide studies have discovered candidate genes and variants associated with lean body mass, muscle weakness, and sarcopenia, revealing insights into the genetic factors contributing to the disorder.

In conclusion, this review synthesized the complex relationships between LBW and sarcopenia. Given the complex character of sarcopenia, we emphasize the importance of interdisciplinary research encompassing genetics, epigenetics, developmental biology, and ageing studies. While our findings contribute to the understanding of these connections, further research is necessary to identify precise pathways and interactions, which will facilitate the development of effective preventive and treatment strategies for this significant public health concern.

## Conflicts of interest

The authors declare no conflict of interest. The funders had no role in the design of the study; in the collection, analyses, or interpretation of data; in the writing of the manuscript; or in the decision to publish the results.
